# Exploring virtual reality object perception following sensory-motor interactions with different visuo-haptic collider properties

**DOI:** 10.1038/s41598-024-59570-x

**Published:** 2024-05-01

**Authors:** Matteo Girondini, Massimo Montanaro, Alberto Gallace

**Affiliations:** 1 Department of Psychology, University of Milano-Bicocca, Piazza dell’Ateneo Nuovo, 1, 20126 Milan, Italy; 2 Mind and Behavior Technological Center, University of Milano-Bicocca, Milan, Italy; 3 MySpace Lab, Department of Clinical Neuroscience, University Hospital of Lausanne, Lausanne, Switzerland

**Keywords:** Psychology, Human behaviour

## Abstract

Interacting with the environment often requires the integration of visual and haptic information. Notably, perceiving external objects depends on how our brain binds sensory inputs into a unitary experience. The feedback provided by objects when we interact (through our movements) with them might then influence our perception. In VR, the interaction with an object can be dissociated by the size of the object itself by means of ‘colliders’ (interactive spaces surrounding the objects). The present study investigates possible after-effects in size discrimination for virtual objects after exposure to a prolonged interaction characterized by visual and haptic incongruencies. A total of 96 participants participated in this virtual reality study. Participants were distributed into four groups, in which they were required to perform a size discrimination task between two cubes before and after 15 min of a visuomotor task involving the interaction with the same virtual cubes. Each group interacted with a different cube where the visual (normal vs. small collider) and the virtual cube's haptic (vibration vs. no vibration) features were manipulated. The quality of interaction (number of touches and trials performed) was used as a dependent variable to investigate the performance in the visuomotor task. To measure bias in size perception, we compared changes in point of subjective equality (PSE) before and after the task in the four groups. The results showed that a small visual collider decreased manipulation performance, regardless of the presence or not of the haptic signal. However, change in PSE was found only in the group exposed to the small visual collider with haptic feedback, leading to increased perception of the cube size. This after-effect was absent in the only visual incongruency condition, suggesting that haptic information and multisensory integration played a crucial role in inducing perceptual changes. The results are discussed considering the recent findings in visual-haptic integration during multisensory information processing in real and virtual environments.

## Introduction

Multisensory experiences characterize our daily life sensory-motor interactions and cognitive processes^1,2^. The surrounding environment engages several sensory modalities simultaneously^3^. Despite the presence of different brain areas involved in processing specific sensory modalities (i.e., occipital cortex for visual stimuli; parietal cortex for tactile stimuli and so on), perception is often characterized by a multisensory unified experience^4,5^, whereas the brain integrates multiple information to provide the most reliable representation of the external world. Specific mechanisms allow us to integrate different inputs in a unitary perception according to required rules (e.g., spatial and temporal constraints^4,6^, but see also^7^ for a discussion about spatial rules in multisensory integration). Among different multisensory experiences, our sensory-motor interactions with objects are often based on visual and haptic signals^8–10^. While vision provides us with a comprehensive understanding of an object's appearance and spatial relationship, haptic perception (the active use of touch; see^11^) involves sensory information captured by human somatosensory and proprioceptive systems, such as tactile, temperature, force-feedback, vibration, texture, muscle tension, and pressure through the body. Visuo-haptic integration is, therefore, characterized by the binding of visual and haptic inputs, and it is fundamental for making our body interact with the environment and with tangible objects.

Considering some simple tasks (e.g., grasping, writing, manipulating objects), we immediately understand the need to integrate visual and haptic information to perform daily activities accurately. However, it is important to note that visual and haptic inputs provide information through different channels, fashions, and processing occurring in different brain areas and circuits^10,12^. Therefore, our perceptual system needs to integrate these incoming sensory inputs to form a unified representation of the physical environment as it interacts with the body. While our ability to integrate diverse sensory information is typically efficient, it's intriguing to note that signals coming from one sensory source often affect the processing of information coming from other sensory sources and thus affect the whole multisensory binding^2,13^.

In a seminar paper, Rock and Victor demonstrated that altering the visual characteristics of objects can impact the haptic perception of that object^14^. Using an optical distortion, the authors induced a mismatch between visual and haptic information of object shape. Participants simultaneously interacted with the object using vision and haptic to judge the object's shape. The multisensory conflict between visual and haptic information was resolved completely in favor of vision, and the haptic experience changed accordantly to the visual inputs provided in each trial. Further studies confirmed the prevalence of visual information on haptic, as demonstrated by Ernst & Banks' works^2^. In a psychophysical study, the authors showed that size perception was mainly driven by vision rather than haptic (*visual capture*) under normal visual-haptic conditions. However, as the overall level of noise in the visual stimulus increased, the weight associated with each modality changed, and participants showed a reverse effect, for which haptic information dominance on vision (e.g., *haptic capture*) was reported. These two relevant studies exemplify the growing body of scientific investigation into the role of visual and haptic cues in shaping our perception of objects (see^10^ for reviews on visuo-haptic integration in object perception).

Nowadays, virtual reality (VR) seems to be a promising tool for investigating multisensory integration due to the feasibility of manipulating sensory information^15^. VR refers to a simulated digital environment generated by computer technology and experienced by users through specific interfaces able to reproduce sensory stimuli such as sight, sound, and sometimes touch. This immersive environment aims to replicate real-world scenarios or create entirely new ones, allowing users to interact with and navigate through this digital environment as if they were physically present (it is worth noting that there are several definitions of virtual reality in the literature. Therefore, we decided to adopt the one described in the text. For a full account of up to 52 different definitions of virtual reality, see the book ‘The down of the new everything’ by Jaron Lanier^16^). Importantly, VR facilitates the dissociation and control of sensory stimuli while maintaining realistic and immersive experiences, rendering it particularly valuable for probing visual and haptic manipulation in human perception. In summary, VR allows increasing the range of manipulations available for testing beyond what can be achieved by using some classic laboratory settings (e.g., the body in VR can rapidly move towards new locations using ‘teleportation’, a way of changing spatial properties of objects that have not been developed in the physical world—at least beside quantum scale).

For instance, Buckingham recently replicated the size-weight illusion (SWI) using virtual reality^17^. In a classic SWI paradigm, participants evaluate the weight of two different objects by lifting them in a sequence. The critical condition to induce the SWI is when two objects with the same mass, but different visual sizes are presented. Despite the same weight, participants often reported the smallest object as heavier than the biggest, reflecting the relationship we experience daily between size and weight, for which size positively correlates with weight^18–20^. Replicating the size-weight illusion (SWI) in virtual reality (VR) offers the advantage of untangling the roles of visual and haptic cues in inducing the illusion through separate manipulation of these components. The potential of VR also relies on the possibility of creating experimental conditions that are impossible (or particularly challenging) to be created in real-life contexts. Ban and colleagues used a visuo-haptic VR (video-see-through system using monitors) system to manipulate the user's hand size perception while participants held objects^21^. It is worth noting that in real interactions, hand dimension can be manipulated by means of a magnifying glass. However, the same manipulation would also increase the size of objects (something that can be instead dissociated with VR). This manipulation generated a spatial nonconformity between visual and haptic feedback received during the interaction. Results showed that participants reported holding an object that did not respect the real haptic features of that object but rather corresponded to what they saw on the visual monitor. Such incongruency was resolved through a *visual capture* of object size perception, altering the participants’ haptic perception. Furthermore, consistently with the idea of a visual capture of tactile sensations, Bergstrom and colleagues demonstrated that it is possible to create an illusion of a bigger or smaller cube also by manipulating the user's finger position (so-called *resized grasping illusion*) while grasping a physical object represented using an immersive virtual environment (IVEs) (using head-mounted display, or HMD)^22^. In a different study based on IVEs, Choi and colleagues combined visual-haptic illusion and active transient vibration feedback to manipulate the objects' softness perception in a virtual environment^23^. Participants interacted with real objects presented in the virtual environment and evaluated softness. Vibration feedback actively modulated the perception of the softness of objects, and the perceived softness of visually observed objects was influenced based on the frequency of the vibrations. In these studies, by means of (immersive or semi-immersive) virtual simulations, the visual and haptic properties were intentionally manipulated, aiming to assess how object perception may be influenced by deliberate adjustments to the sensory information provided to participants.

Nevertheless, VR presents some limitations in simulating visual-haptic interactions as they could appear in real laboratory contexts. It's important to acknowledge that the complexity of visual-haptic interactions limits the possibility of recreating simulations of body-object interactions as they occur in reality. For instance, force feedback (allowing to reproduce also proprioceptive information regarding the force excerpt by our body muscles on the object to be manipulated) can be achieved only using selected devices that artificially try to mimic the weight or inertia feedback during virtual interactions; however, these devices are often complex to use, with limited workspace (and degree of freedoms of the exploration movements allowed), and are expensive compared to the cost of commercial VR headsets^24^.

Furthermore, the precise design of visual-haptic objects and body parts within virtual environments is crucial to maintaining consistency between the multisensory experiences encountered in reality and those simulated virtually. As far as this point is concerned, Lougiakis and colleagues compared the participants’ performance in a visuomotor task (moving a virtual object toward a destination point) by using three different visual representations of the arm effector: a virtual sphere, a VR controller, and a virtual hand^25^. Under the sphere condition, the participants performed worse than the other two conditions. However, no differences were found between the VR-controller and the hand representations (although participants reported a stronger sense of ownership in the latter case), and the lack of benefit in task performance using a virtual hand has been interpreted as a mismatch in virtual hand behavior compared to those in the real world. Venkatakrishnan and colleagues' study further confirmed these findings by comparing the efficacy of arm effectors represented through a VR controller versus controller-hand representation^26^.

As far as the importance of different sensory cues in virtual interactions is concerned, De Siquera and colleagues employed a VR environment to study how participants perceive the size of virtual objects within their reachable space under different conditions: visual, haptic, or visuo-haptic presentation^27^. Their findings mirrored real-world situations, showing that participants tended to overestimate size when relying solely on haptic feedback, compared to when both visual and haptic feedback were available^28^. However, participants exhibited better performance in size estimation under the visual-only compared to the visual-haptic condition, resulting in higher accuracy in size perception with unimodal (visual) than bimodal (visual and haptic) exploration. Once again, the authors attributed this result to a conflict between non-ecological visual and haptic interaction within the virtual simulation. All of the above-mentioned studies raise an interesting point about how our perceptual system responds to sensory stimulation (visual or visual-haptic), when it does not properly respect the rules of physical words, something that can occur in virtual interactions involving visual and haptic information.

Overall, previous studies suggest the pivotal role of both visual and haptic information in shaping object perception during our interactions with the world. Moreover, they also suggest that our final perceptual outcome would seem to be the results of spatio-temporal contingencies (experienced during our lifetime) between visual and haptic object proprieties of our environment. Given that VR enables the manipulation of such contingencies, our interest was directed to understanding how changing the parameters of the visuo-tactile interaction with an object can lead to temporary changes in object perception.

As far as this point is concerned, it has been demonstrated that manipulating visual and haptic information during object interaction can lead to alterations in object perception, resulting in immediate perceptual changes (referred to as online effects)^2,14^. However, being exposed to specific unisensory and multisensory conditions for a prolonged period (i.e., changing sensory contingencies) can also modify object perception in subsequent phases (known as perceptual after-effects), leading to adaptive perceptual processes. Surprisingly, research on the potential after-effects of prolonged exposure to specific conditions of visual-haptic stimulation during object manipulation in VR, particularly concerning size perception in object perception, is poorly investigated. This research question is relevant for at least two aspects previously introduced. The first pertains to the importance of designing suitable multisensory experiences that integrate visual and haptic elements in virtual interactions. As immersive virtual environments are increasingly employed for training simulations of real-world scenarios, ensuring appropriate conditions becomes essential to maintain the ecological validity of these simulations. Indeed, virtual environments frequently necessitate prolonged periods of object manipulation and interaction, including the use of tangible virtual objects within the simulation^26,29^. Secondly, particularly pertinent to the present study, a discrepancy in sensory information during object interaction within a virtual environment could lead to perceptual after-effects that can be representative of a change in our representations of the world. That is, this phenomenon might represent a recalibration in object perception, influenced by the visual and haptic features experienced during prior interactions. It is worth noting that no study in real contexts so far has completely separated the role of visual and tactile attributes involved in size perception following active sensory-motor interactions (the natural way in which associations between visual and tactile aspects of an object are determined). In fact, in order to achieve this, one needs to visually touch an object (to determine its size) without feeling touch at such a point of contact (but feeling it before entering the object contour—bigger tactile size—or after—smaller tactile size). This can be easily done in VR using the size of a collider (e.g., the invisible space that triggers a response from the environment; in this case, the activation of tactile feedback), but it can become much more complicated to be achieved in the real world (e.g., perhaps using extremely precise flows of air generated by the point of contact or magnetic fields). The impact of visual-haptic interaction with a virtual object on its perception, particularly regarding size discrimination, remains thus unclear. In this context, immersive virtual environments offer us the chance to easily control and manipulate visual and haptic aspects of sensorimotor interactions and evaluate how these elements affect size perception by observing changes in performance occurring after the interaction. Prior investigations suggest that humans are able to distinguish tiny differences in object size when presented using VR^30^. Moreover, in a previously conducted preliminary study, we demonstrated that manipulating the collider of a virtual object (responsible for detecting collisions and interactions within the virtual environment), coupled with haptic feedback (vibration), caused a distortion in the perception (as a form of perceptual after-effect) of the virtual object's size^31^. However, given the preliminary nature of the study, we were not able to disentangle the role of visual to haptic manipulation in inducing this illusion. Here, we present a larger investigation involving different cohorts that could interact with the virtual object presenting a normal or smaller collider, with the presence or absence of haptic feedback during the interaction, measuring object size perception before and after the interaction.

The primary objective of the current study is to determine whether the perceived size of a (virtual) object can be influenced by the visual and haptic features that characterize a prior phase during which the participant interacts with the same object (i.e., the creation of new sensory contingencies). To address this question, we developed a virtual reality paradigm that involved a size discrimination task (using the method of constant stimuli) between virtual cubes of different sizes. Between the two measurements of object size perception (occurring at the beginning and end of the experiment), participants performed a visuo-motor-tactile task (manipulation task) involving interaction with the same cube for 15 min (pushing the cube with a virtual stick towards a final position). To study potential after-effects resulting from cube interaction's visual and haptic features, we divided participants into four groups, each interacting with a cube characterized by different visual and haptic attributes. Specifically, the collider of the cube (i.e., the interactive invisible space that surround an object in virtual reality and determines its behavior when it collides with other objects) was manipulated with respect to the (virtual) physical boundaries of the cube, which could either adhere to or deviate (using a smaller collider) from the visual boundaries of the cube. To further disentangle the relevance of haptic information, participants could receive or not (depending on the experimental groups in which they are involved) haptic feedback (through vibration) from the hand when the stick collapsed with the collider (either inside or at the boundaries of the cube). In accordance with our previous result, we hypothesized the presence of perceptual after-effects in the size discrimination task in the groups that interacted with the small collider, which was not present in the control groups (normal-sized collider). Moreover, we thought that the presence of vibrations could potentially increase perceptual after-effects by reflecting multisensory additivity properties of visual manipulations. Lastly, we anticipated difficulties in interacting with the cube surrounded by the small collider during the manipulation task as a form of unfamiliar sensorimotor interaction, indexed by the total number of trials completed in a fixed amount of interaction time.

## Methods

### Participants

A total of 100 (Female = 62) participants aged 18–42 y.o. (M = 23.2, SD = 4.8) were recruited by self-enrollment using the University recruiting platform. The sample size was chosen a priori and calculated using G* Power 3.1 (beta = 0.9, alpha = 0.5, small effect size 0.2), which resulted in a total sample of 25 participants for each group. After recruitment, four participants were excluded due to technical problems (n = 2) or reversed responses (n = 2). A total sample of 96 participants was included in the final analysis. The protocol of this study was approved by the Ethical Committee of the Department of Psychology of the University of Milano-Bicocca and conducted following the standards of the Helsinki Declaration.

### Experimental design

The experimental design involved participants performing two size-discrimination tasks before and after a manipulation task. Each participant performed the categorization task—manipulation task—and categorization task in this order. During the categorization task, two cubes were presented sequentially: the first (reference cube) always had the same size, while the second cube differed in size in each trial. Participants were required to indicate if the second cube was bigger than the first (see *Categorization task)*. Between the two size-discrimination measurements, they performed a manipulation task in virtual reality, in which they had to interact by using a virtual stick with a virtual cube (the same as the one adopted in the categorization task) to complete a visual motor task. During this phase, participants moved a virtual cube using two virtual sticks from an initial (spawning) position to a random target point in the virtual environment (a simple room) (see *Manipulation task)*. All groups performed the size discrimination task before and after the manipulation task. Two elements of the cube were manipulated: a) the size of the collider (point of interaction) between the stick and the cube and b) the presence of haptic feedback (vibration) during the stick-collider collision. More specifically, we manipulated the visually perceived point of interaction with the object (collider coincident or smaller than the visually perceived size of the cube) and the presence of haptic feedback resulting from the interaction.

Participants were distributed in four experimental groups:

- *Normal Haptic (N* = *25)*: The interaction was composed of a visual normal collider and a vibration every time the stick collapsed with the collider
- *Normal No haptic (N* = *22)*: The interaction was composed by a visual normal collider, but no vibration was provided when the stick collapsed with the collider
- *Small No haptic (N* = *24)*: The interaction was composed by a visual small collider, but no vibration was provided when the stick collapsed with the collider
- *Small Haptic (N* = *25)*: The interaction was composed of a visual small collider and a vibration was provided every time the stick collapsed with the collider

**Figure 1:**
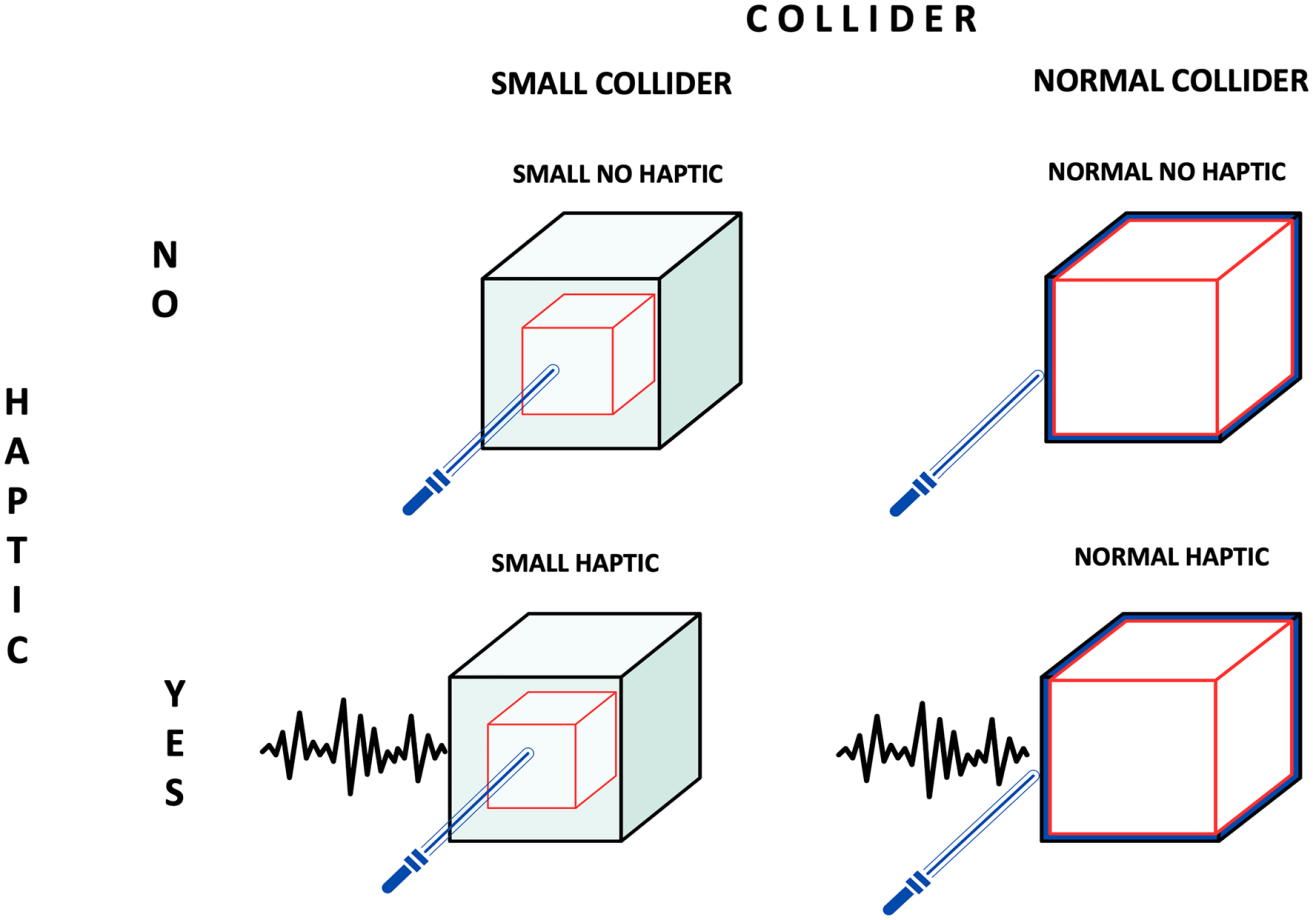
This visual representation illustrates the experimental setup used in the study. On the left is a depiction of the small collider (highlighted in red), while the right side shows the normal collider. The top row showcases interactions between a stick and a cube without haptic feedback, whereas the bottom row shows groups that interacted with haptic feedback.

Each group interacted with a specific cube, resulting from a different combination of visual and haptic features (Fig. 1). Specifically, the virtual collider could be characterized by a normal vs. small *visual collider* and the presence of vibration vs. non-vibration *haptic feedback* during the interaction (see Fig. 1 for a graphical representation of experimental conditions). This design resulted in a 2 × 2 × 2 mixed design, with the main factor of *time* (pre vs. post) as within participants and *collider* (normal vs. small) and *haptic* (haptic vs. no-haptic) as between factors.

### Hardware and software

The VR equipment used for the experiment included an Oculus Quest2 Head mounted display (HMD), with a 1280 × 1440 pixel resolution per eye (refresh rate 80 Hz). The HMD was connected to a computer, featuring an Intel Core i7-7800X CPU, 16 GB of RAM and a GeForce GTX GPU.

Two VR environments were developed with the Unity graphics Engine (https://unity.com/.) for the purposes of this experiment: The categorization task and the manipulation task virtual environments. An empty room was used as the virtual space for both conditions.

### Categorization task

**Figure 2:**
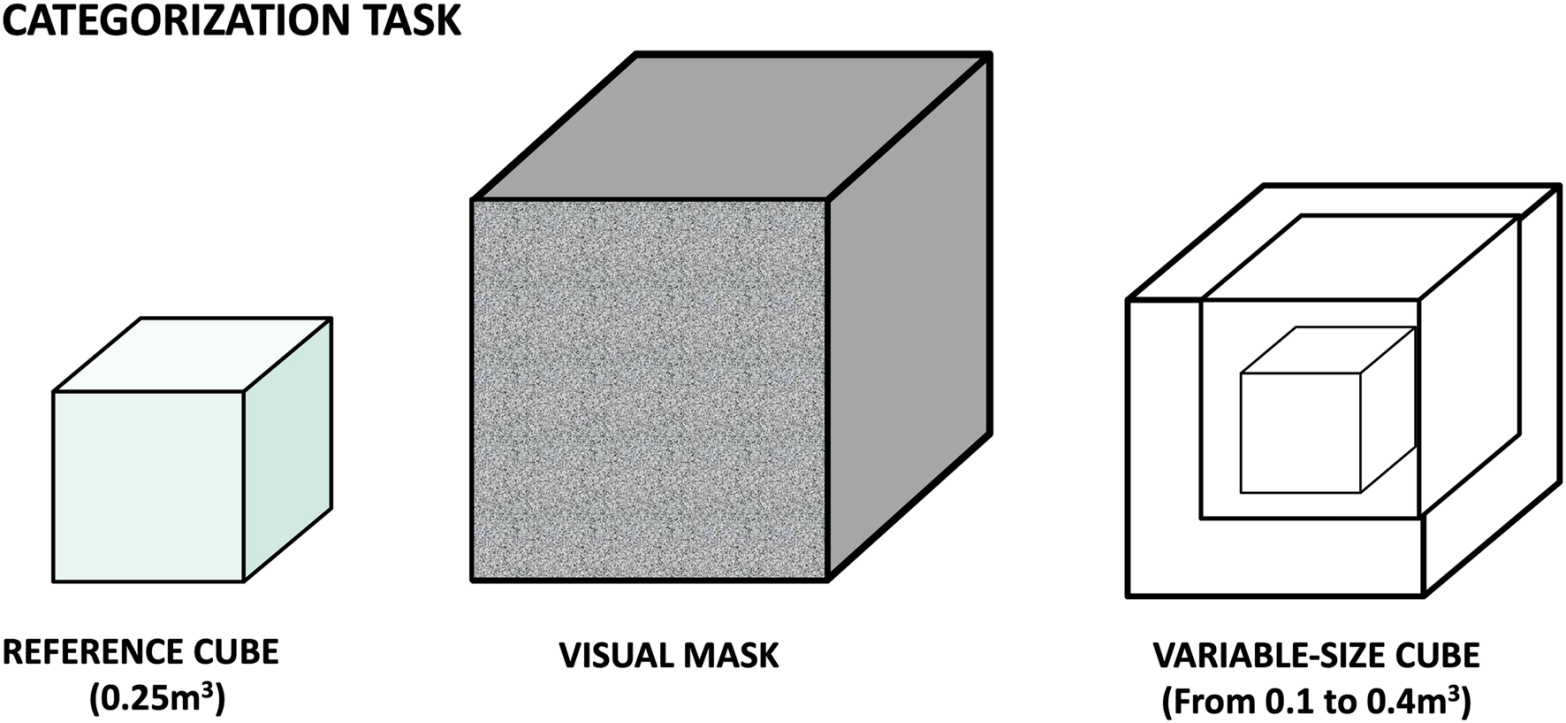
Example of categorization trial: The first cube always had the same size (0.25). A visual mask cube follows it, and then the second cube appears. The second cube changed size in each trial. Participants had to indicate if the second cube was bigger or smaller than the first one, using the right (yes) or left controller (no).

During the categorization task, a sequence of two different cubes was presented to the participant. The first cube (reference cube) had a fixed size (0.25 m^3^), while the second cube (variable cube) varied its size in each trial, ranging between 0.10 and 0.40 (0.10; 0.16; 0.22; 0.28; 0.34; 0.40 m^3^). A visual cubic mask was presented between the first and second cubes (0.75 m^3^). The participant had to evaluate (forced choice in a fixed window of 4 s) if the second cube was bigger (or not) than the reference cube, using the VR-controllers to provide a response (left click for “small” response and right click for “big” response). Each block contains 63 trials (each comparison repeated 9 times). The order of trials was randomized across participants. Note that the reference cube used for the categorization task was the same as the manipulation task (indexed by the same color) (Fig. 2).

### Manipulation task

**Figure 3:**
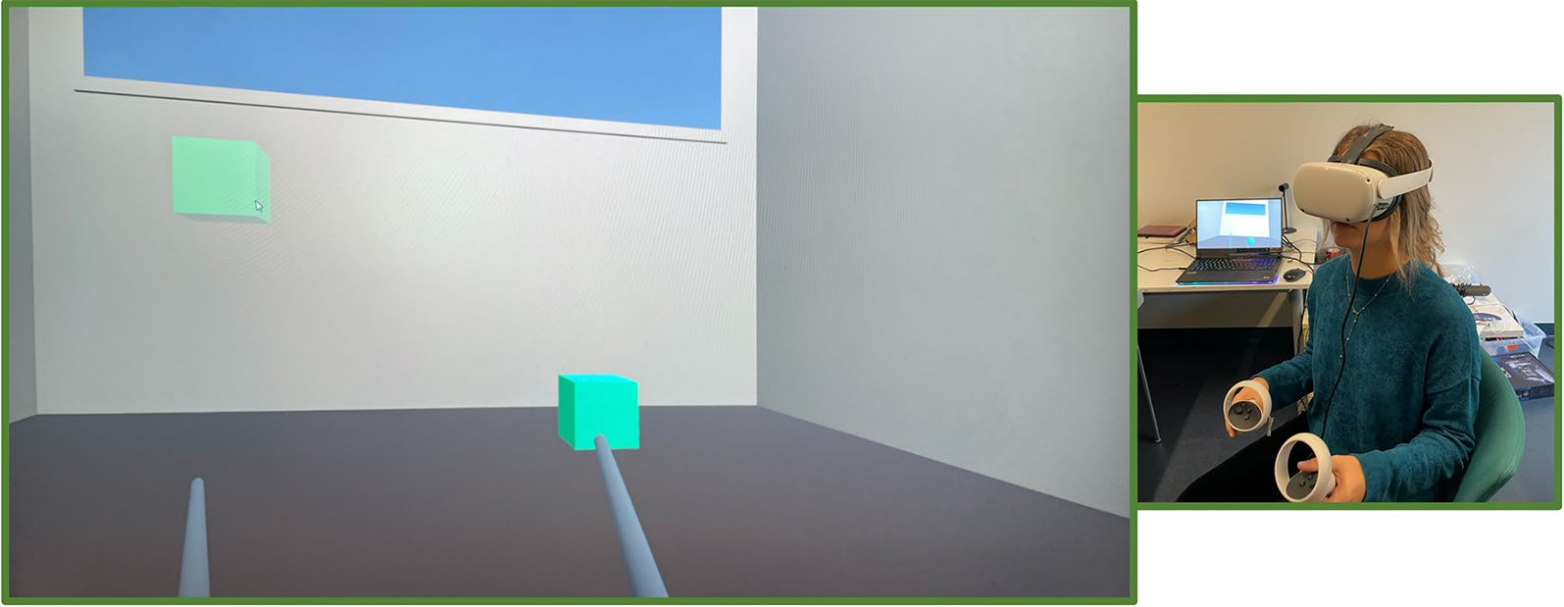
Example of manipulation task of small collider condition. The interaction between the stick and the cube appeared inside the visual boundaries of the cube. On the right side, the participant during the manipulation task.

The manipulation task lasted 15 min, and participants interacted with a virtual cube using their right and left hands. The goal of the task is to move the cube with the two virtual sticks (controlled through the headset's proprietary controllers) to fill it in a destination point (Fig. 3). At the beginning of each trial, a virtual cube (the target cube) appeared in the center of the virtual environment, while a semi-transparent cube appeared randomly in one of four locations in the virtual room (bottom-left, bottom-right, upper-left, upper-right). Each participant received the same instruction: touch and move the target cube using the virtual stick to match its position with the semi-transparent cube's. Once the target cube reached the semi-transparent cube, the trial ended, and a new target cube appeared in the center of the virtual room. Each group constantly interacted with the same virtual cube, which was characterized by specific visual and haptic features in accordance with the assigned experimental group.

### Measurements

The outcome measurement for the size discrimination task was the participants' point of subjective equality (PSE). PSE represents the stimulus level at which the participant perceives two stimuli as equal or identical regarding a specific attribute or characteristic, and it provides insights into participants' perception accuracy and discrimination ability in the size discrimination task^32,33^. Specifically for this experiment, PSE was used to evaluate the presence of bias in size perception for a virtual cube.

The outcome measurement for the manipulation task concerned participants' performance, which was measured by the number of touches in each trial and the total number of cubes performed in 15 min.

### Procedure

**Figure 4:**
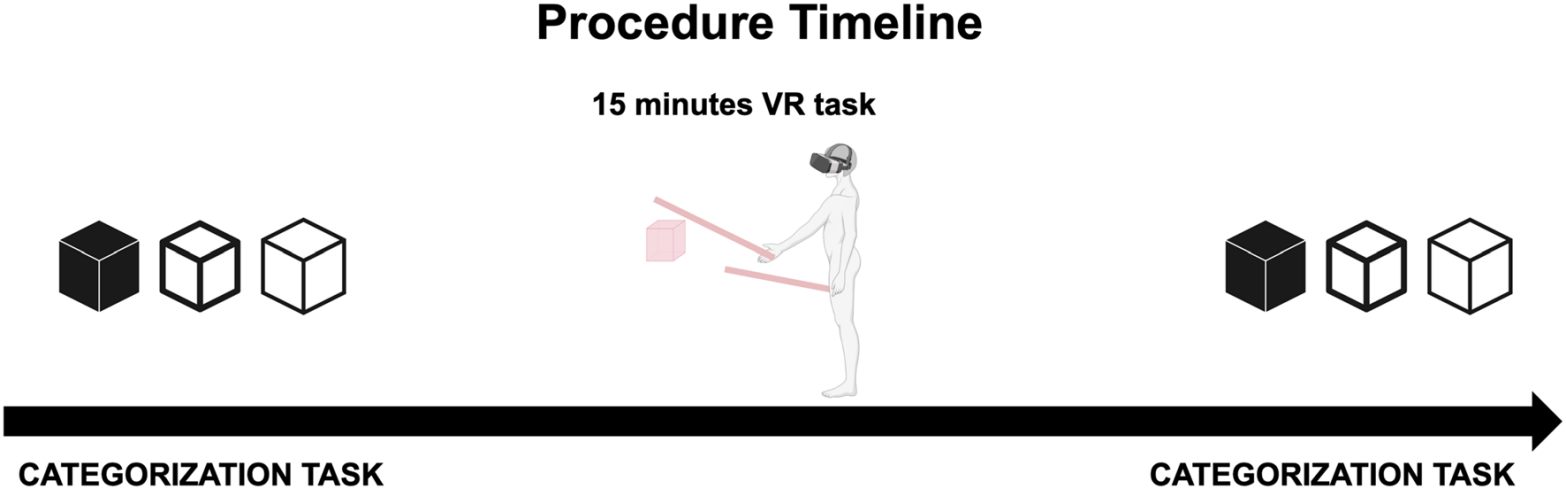
Experiment timeline.

The participants arrived at the laboratory and signed the informed consent form. The experimenter explained the different stages of the experiment and the instructions for each task. The experiment was a fixed block design, for which the participant started performing the baseline categorization task, then 15 min of manipulation task, and concluded with the categorization task post-manipulation. When everything was clear, the participant wore the HMD and took the controllers. A training phase was included before the real testing to ensure the correct understanding of the tasks. In this phase, the participant performed 1 min of the categorization and 1 min of the manipulation tasks. Data from the training phase was not included in the analysis. After performing the three experimental blocks (categorization-manipulation-categorization), participants removed the HMD, and the experimenter provided a brief description of the aim of the study and experimental manipulation. Figure 4 shows a graphical representation of the experiment timeline.

### Data analysis

Data analysis was performed using R (www.r-cran.com) and R studio. We extract the psychometric curve for each participant in the size discrimination task. The logistic regression model measured the point of subjective equality for each participant at each time (pre vs. post). The PSE values were submitted to a 2 × 3 mixed model ANOVA, with *time* (pre vs. post) as within condition and collider (*normal vs. small)* and haptic (*vibration vs. no-vibration)* as between factor design. In case of a significant interaction effect, the planned analysis compared differences in PSE between pre and post-measurement among different groups, using paired sample t-test corrected for multiple comparisons (Bonferroni correction for multiple comparisons). For the manipulation phase, the number of touches in each trial and the total amount of trials completed in 15 min were extracted and analyzed using 2 × 2 mixed model ANOVA, with collider (*normal vs. small)* and haptic (*vibration vs. no-vibration)* as between factors. Significant p-value was set to 0.05, and generalized eta square (ω2) was reported to measure the effect size.

## Results

### Manipulation task

**Figure 5:**
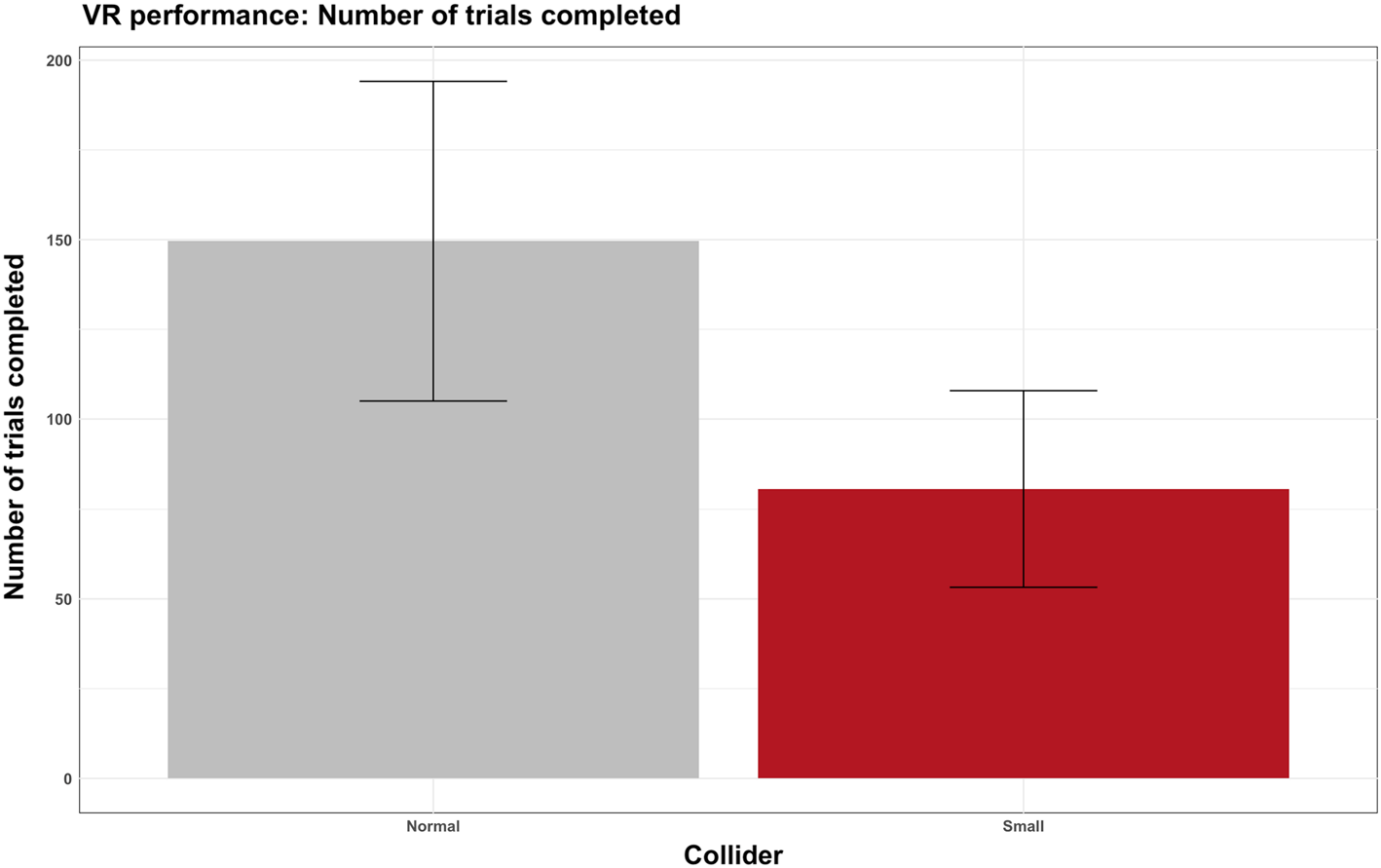
Plot of manipulation results: the number of trials performed with the normal collider versus the small collider. Error bars represent standard deviation.

A main effect of collider (F = 82.74, df = 1,92, p < 0.001, ω2 = 0.473) revealed that groups that interacted with the small visual collider completed fewer trials (M = 80.6, SD = 27.3) compared to normal collider (M = 150, SD = 44.5). No main effect of haptic (F = 1.48, df = 1,92, p = 0.226, ω2 = 0.01) or interaction collider * haptic (F = 0.24, df = 1,92, p = 0.622, ω2 = 0.001) were found (Fig. 5).

**Figure 6:**
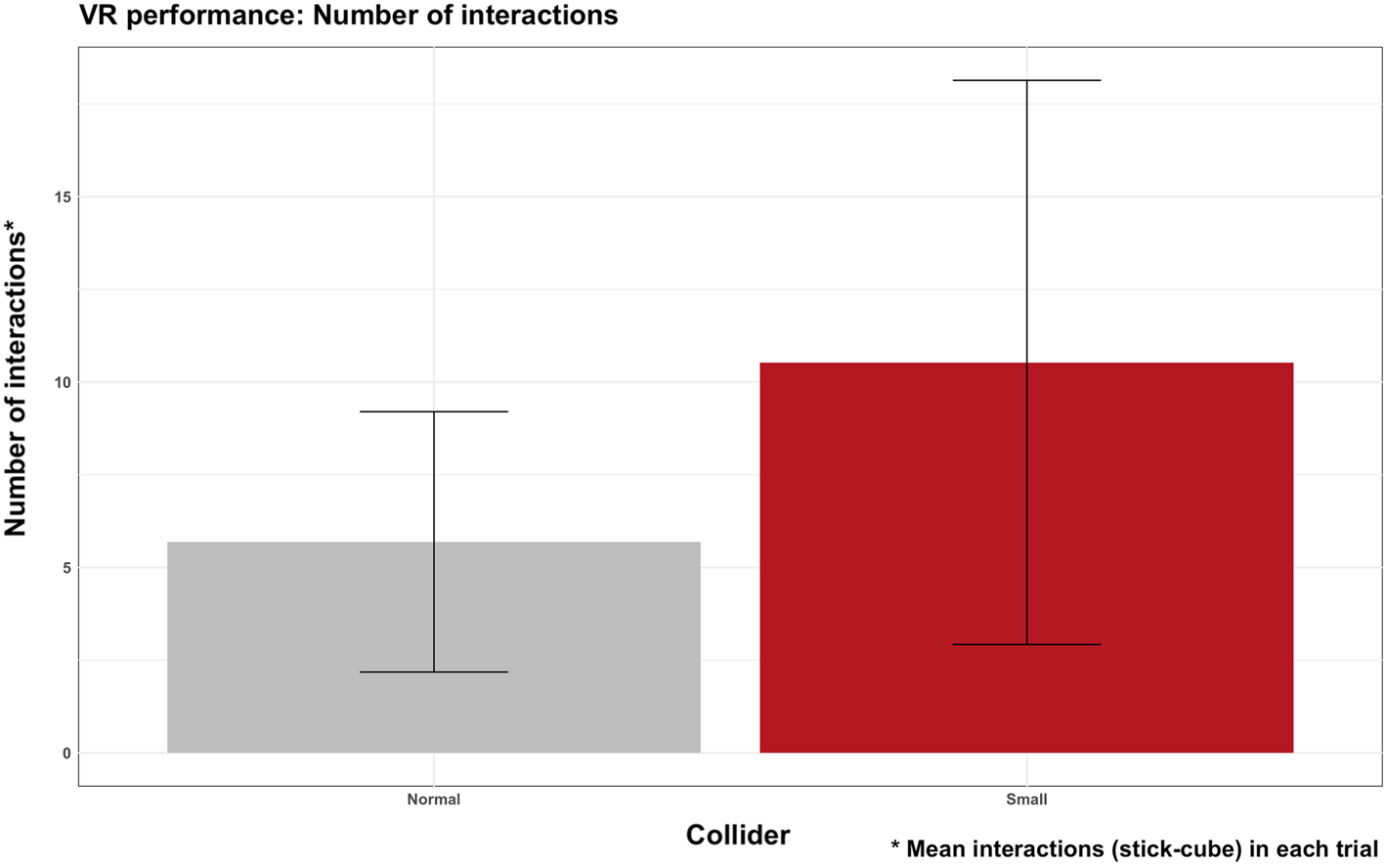
Plot of manipulation results: the mean number of interactions performed with the normal collider versus the small collider. Error bars represent standard deviation.

Similar results were found in the number of interactions in each trial. A main effect of the collider (F = 15.24, df = 1,92, p < 0.001, ω2 = 0.142) was found regarding the number of interactions performed with the cube to complete each trial. Interacting with a small visual collider required more touches (M = 10.5, SD = 7.55) to complete the trial than the normal visual collider (M = 5.6, SD = 7.55). No main effects of haptic (F = 0.26, df = 1,92, p = 0.608, ω2 = 0.003) or interaction collider * haptic (F = 0.06, df = 1,92, p = 0.806, ω2 = 0.001) were found (Fig. 6).

### Categorization task

**Figure 7:**
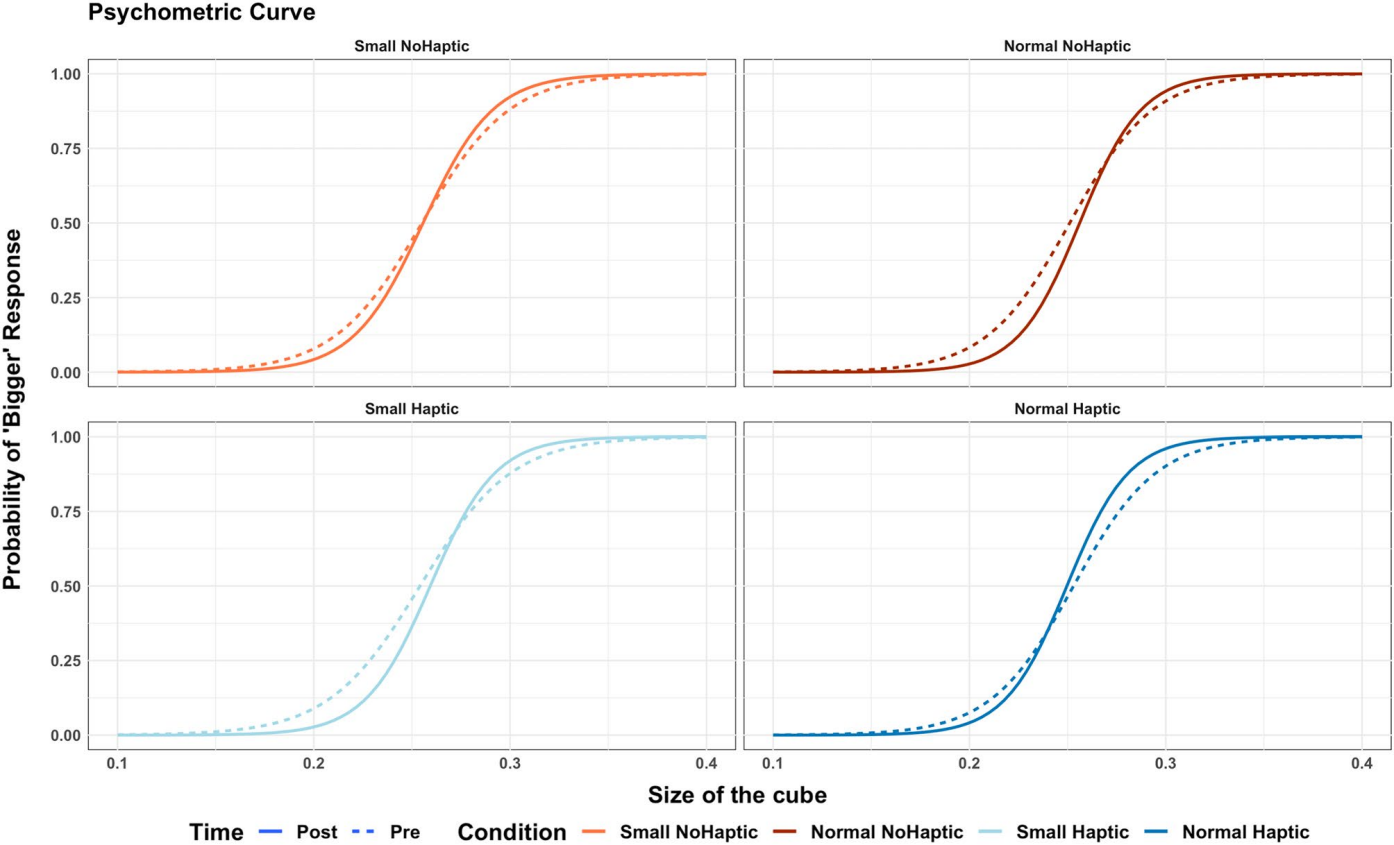
Grand average of psychometric function in the four groups. The dashed line represents the baseline measurement, and the continuous line represents the post-VR measurement.

The grand average of the psychometric function between the four groups is represented in Fig. 7.

**Figure 8:**
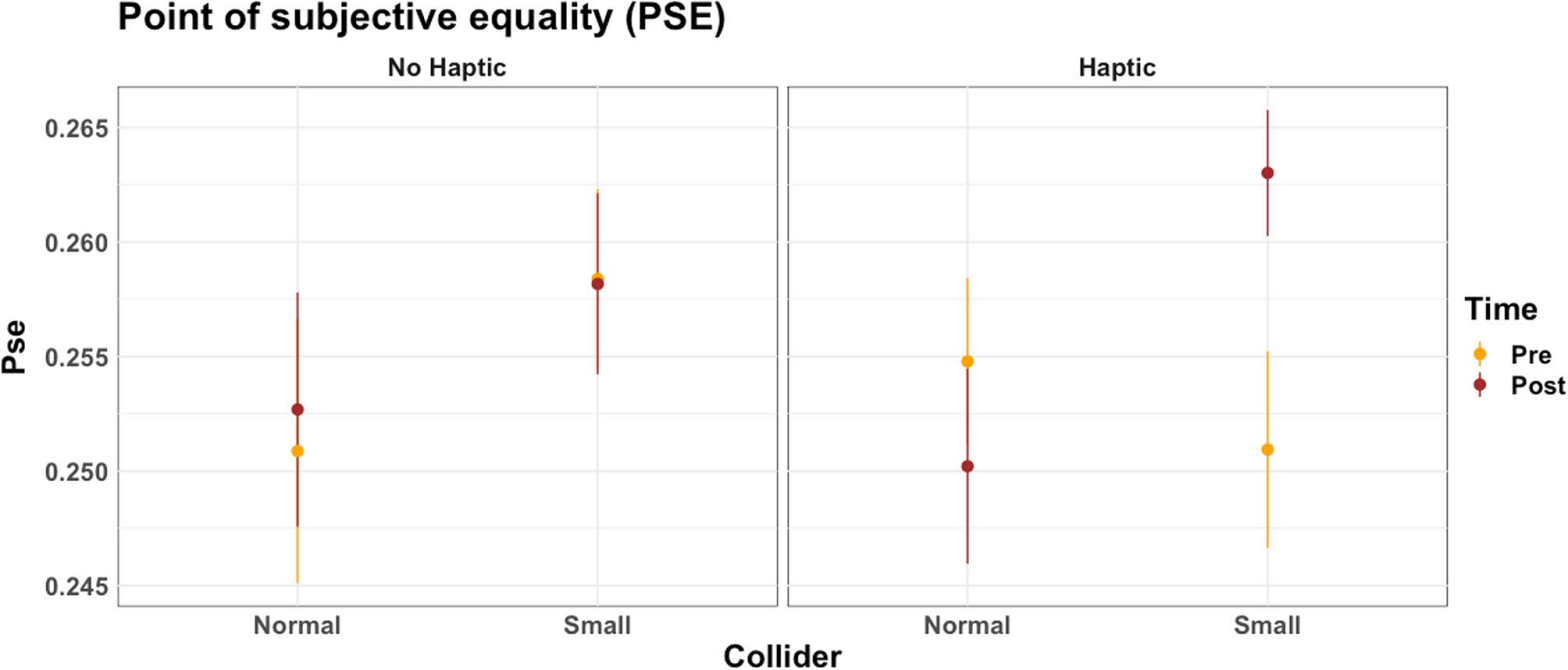
Mean value of the point of subjective equality. On the x-axis, the visual collider (small vs. normal). On the y-axis is the mean value of PSE. Yellow dots represent the PSE at the baseline; red dots represent the PSE after the VR task. On the left side are the groups with haptic stimulation. On the right side, the groups without haptic stimulation. Error bars represent standard deviation.

*Point of subjective equality*: No main effect of the time (F = 1.32, df = 1,92, p = 0.253, ω2 = 0.003), collider (F = 2.14, df = 1,92, p = 0.146, ω2 = 0.017), or haptic (F = 0.07, df = 1,92, p = 0.932, ω2 = 0.001) was found regarding the point of subjective equally. The two-way interactions collider * haptic (F = 0.07, df = 1,92, p = 0.786, ω2 < 0.001) and haptic * time (F = 0.61, df = 1,92, p = 0.434, ω2 = 0.001) were not significant. However, the interaction collider * time was close to reaching a significant effect time (F = 3.66, df = 1,92, p = 0.058, ω2 = 0.009). Critically, the three-way interaction time * collider * haptic was significant (F = 5.37, df = 1,92, p = 0.022, ω2 = 0.013) (Fig. 8).

**Figure 9:**
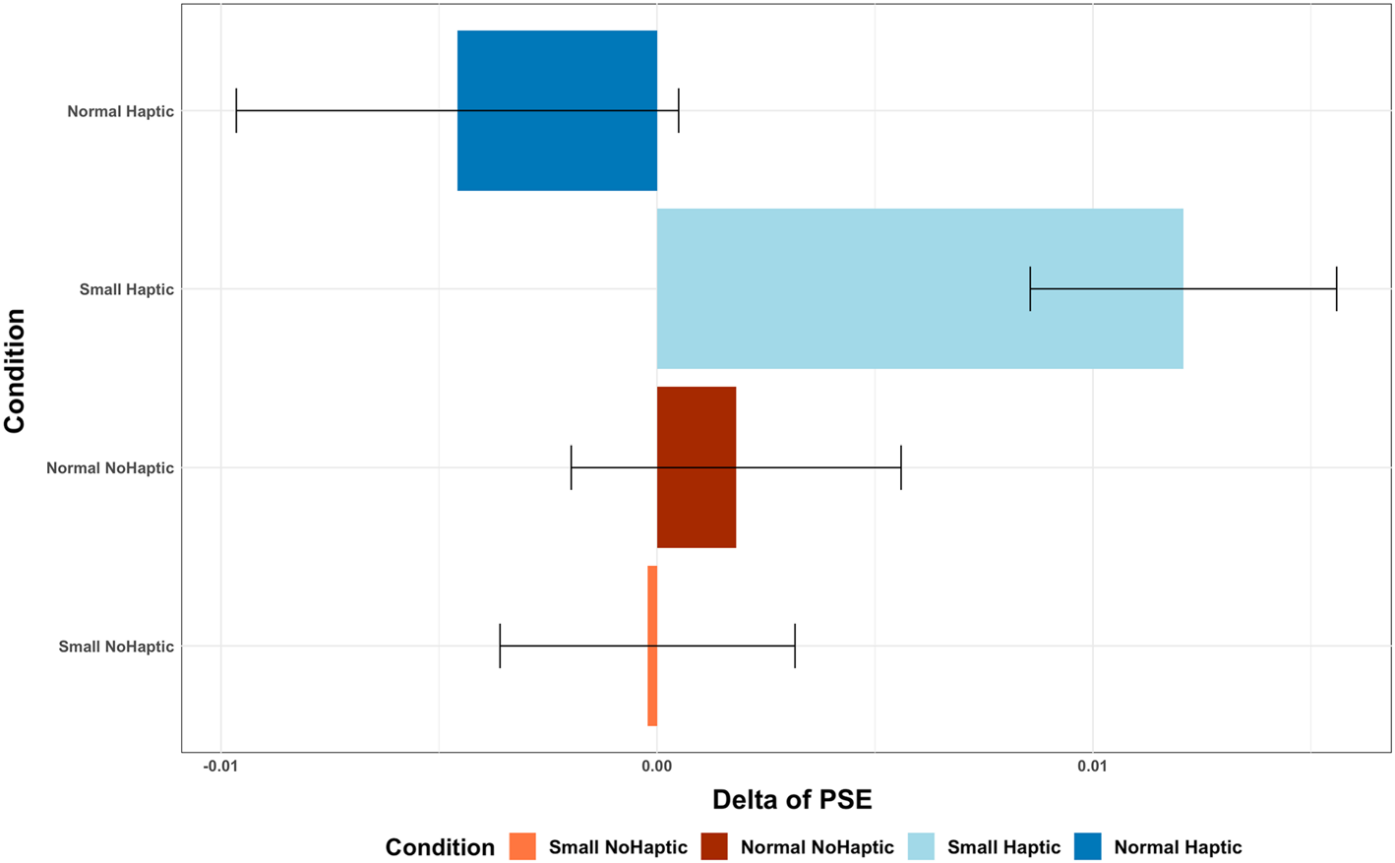
The delta difference in PSE (PSE baseline—PSE after VR) was calculated for each of the four groups. Positive values indicate that the cube was perceived as larger after the virtual reality (VR) experience. Error bars represent standard deviation.

To estimate the source of the interaction, we performed separate paired sample t-tests (Bonferroni correction for multiple comparisons) in each group to measure changes in PSE. Differences in PSE were not significant comparing pre vs. post in the group of Normal Haptic (p = 0.380, *difference*_*mean*_ =  − 0.004), Normal Nohaptic (p = 0.640, *difference*_*mean*_ = 0.001), and Small Nohaptic (p = 0.950, *difference*_*mean*_ =  − 0.001). On the contrary, a significant difference was found when PSE was compared pre vs. post in the small haptic group (p = 0.002, *difference*_*mean*_ = 0.012). The point of subjective equality increased in the post-manipulation measurement (M = 0.263 m^3^, SD = 0.01) compared to the baseline measurement (M = 0.251 m^3^, SD = 0.02). The average delta difference (calculated as PSE_pre_ − PSE_post_) in each group is reported in Fig. 9.

## General discussion

The present study aimed to investigate a possible after-effect in size perception driven by prolonged exposure to a visuo-haptic conflict during object interaction. Participants performed a size discrimination task two times involving the discrimination of size cubes in virtual reality. The task was performed at the baseline and after 15 min of visuo-motor-haptic task (manipulation task) involving an interaction with the same virtual cubes used in the discrimination task. The experimental design included 4 experimental groups, in which the cubes' visual (small vs. normal collider) and haptic (vibration vs. non-vibration) interactive features were manipulated. For the visual component, we manipulated the size of the cube collider, which delineated the area in which the interaction with the stick occurred; note that only within the space of the collider the stick can push the cube. Specifically, the virtual object collider could conform to the cube's shape and size (normal collider) or be contained within its boundaries (small collider). Regarding the haptic component, cube interactions could be characterized by the presence vs. absence of haptic (vibration) feedback. We then compared the participant’s performance in the size discrimination task and during the manipulation tasks (number of touches required to complete the trial, i.e., to push the cube into its final position) across these conditions.

We found that participants who interacted with the small collider completed fewer trials than the neutral collider, regardless of the presence or absence of haptic feedback. Moreover, a small collider required more touches to complete the trial (move the cube from the starting point toward the destination point). This result is in line with the result of our first preliminary study on this matter^31^, suggesting an impairment in performing the manipulation task in case of visual incongruency between the shape of the cube and its interaction area (the collider). This result might represent a signature of the visual capture mechanism during visuomotor interaction in virtual reality. The impairment in the manipulation task might be related to the fact that goal-oriented movements, such as grasping, pinching, or tool manipulation, primarily involved the dorsal visual pathway^34^. The dorsal visual pathway (known as “visual for action” or visuomotor pathway) processes information time by time to complete visuomotor actions toward specific spatial targets, as in the case of external objects^35^, using the metric signals provided by visual information to control and adapt the movement effectors^36^. Compared to the ventral visual pathway (known as vision for perception), which is involved in more structural and complex object representation, the dorsal pathway relies on bottom-up information from vision to specify the movement parameters^36^. This visuomotor pathway strongly relies on visual input to coordinate body effector and movement. During the manipulation phase, the visual constraints of the cube guide the visuomotor movement directed to the virtual object. However, when dealing with small colliders, this led to a conflict between the visually perceived cube borders and the invisible interaction point (only experienced during the contact with the virtual stick), as the cube's visual borders mainly guided the stick movements in space, but the actual interaction was not aligned with them. Here, haptic feedback provided only a confirmatory signal of when the interaction occurred (e.g., we might say that haptic feedback was redundant). During the manipulation phase, performance was notably influenced by the visual incongruence itself, with no discernible differences attributable to the presence or absence of haptic stimulation.

The second relevant result of this study is the shift in the participants’ point of subjective equality (PSE) caused by prolonged exposure to visual-haptic incongruency during the manipulation task. Using a psychophysical approach, it was possible to extract PSE, which represents the higher level of uncertainty (50% to make one choice or the other) in size cube comparisons. We then compared the PSE values across the four groups within the two categorization tasks (before and after the interactions with the virtual cubes). Compared to the baseline, the group who experienced a visuo-motor interaction characterized by visual incongruency (collider smaller than the visual size of the cube) and the presence of haptic feedback showed an increase in the point of subjective equality. More specifically, the participants perceived the reference cube (moved in the previous manipulation task) as bigger than when assessed in the baseline measurement. No other difference compared to the baseline was found in the other groups, suggesting that only the condition where the small colliders were coupled with the presence of haptic feedback, (i.e., the combination of visual collider incongruency and haptic signals), led to perceptual changes in the cube size. One might wonder what caused the changes in size perception found in the small haptic collider. One potential explanation for our results could be related to the interaction features experienced during the manipulation phase. Specifically, the results revealed that a small collider required more touches than a regular/normal collider in order to be accomplished. Perhaps interacting with the small collider led to challenging and less realistic interactions, resulting in participants encountering constant difficulties in moving the cube. Consequently, the difficulty of the interaction caused a perceptual bias toward perceiving the cube as larger. However, this interpretation does not justify why this effect was only present in the small haptic condition, given that impaired performance was not dependent on visual incongruency alone (when the small collider was presented without haptic feedback). Indeed, it is more plausible that adding vibrations as an additional sensory cue during the interaction, amplified the altered perception resulting from the visual incongruence.

In a previous study, visuo-tactile feedback during virtual cube interactions resulted to improve depth perception, suggesting the value of multimodal feedback in virtual reality for constructing a more solid object perception^37^. Moreover, fMRI studies showed that the occipitoparietal cortex, a multimodal visuo-haptic area that responds to objects presented visually or haptically, is involved in a more abstract representation of objects^38^. The fact that in the small collider condition, the area of the interaction was placed inside the visual boundaries of the cube may have resulted in representing the size of the cube as bigger. Notably, in both the haptic groups, vibration feedback was temporally bound with the cube interaction (when the stick collapsed with the collider surrounding the cube), generating a specific multisensory visuo-haptic experience with the cube^6^. This could have potentially enhanced the multisensory illusion of interacting with a smaller cube. The subsequent mechanism derived from the integration of visual and haptic cues (according to multisensory *additivity* properties^39^) might have played a role in perceptual changes regarding the perception of the object itself (bigger than its interactive area). In the context of the present study, introducing the haptic feedback when the stick was inside the cube (visually incongruent interaction) could have thus amplified the alteration of the cube's visual representation. The synchronization of haptic feedback with incongruent visual interactions raises the possibility that haptic feedback might have enhanced a distorted representation of the virtual cubes, thereby inducing changes in their size perception. This may also explain why such an effect was absent in the group that interacted with the small collider without receiving the haptic feedback. However, it is important to note that since the present paradigm introduces a new experience in visual-haptic manipulation (mismatch between physical boundaries and interaction boundaries), that is rarely experienced in real interactions and that we measured perceptual after-effects in virtual reality, interpretations of effects might result difficult. In particular, any theoretical interpretations linking the dimension of the small collider and its effect in changing object size perception or representation (e.g., the relationship between drift in size perception and the dimension of the small collider) should be cautiously approached.

Finally, the after-effect found in this study needs to be discussed in light of previous studies investigating object interaction in virtual reality. Specifically for immersive virtual environments, interaction realism between users and the environment is fundamental to guaranteeing the ecological validity of simulations. Despite the fast progression in developing HMD with increased visual resolution (for instance, increasing the device's refresh rate), realistic interaction toward tangible virtual objects is lacking^40^. Moreover, hand animations, such as grasping or pinching a virtual object, are often unrealistic and exemplified, leading to a mismatch between real-world interactions and virtual simulations. Unfamiliar movement-guided interactions and the restricted simulation of haptic feedback when interacting with virtual objects pose significant challenges in virtual simulations involving object interaction. This discrepancy in the interaction between real and virtual objects may explain the absence of advantages when utilizing a virtual hand mesh compared to using a VR controller as the representative effector reported in the literature^25^. Moreover, it could account for the lack of advantage in visual-haptic perception when discerning the size of tangible objects presented in virtual reality^27^. To date, possible perceptual after-effects driven by inconsistency in sensorimotor object interactions are mainly unknown. Our findings demonstrate that manipulating the multisensory properties of virtual objects during prolonged interaction results in alterations in the perception of their size, highlighting the link between sensorimotor interactions (or contingencies) and perceptual outcomes in virtual simulations.

### Limits of the study

This study still presents some limitations that need to be discussed. First, the haptic feedback provided during the interaction was a vibration, reducing the perception of realism in the scene. In the future, haptics will need to be integrated into virtual environments with proper devices to mimic different (haptic) sensations (for instance, force feedback can activate the proprioceptive system through mechanoreceptors), depending on the characteristics of the interaction (e.g., by using gesture tracking or haptic gloves^24^). Moreover, in our study, a further group in which the haptic condition was also spatially manipulated (e.g., vibration coincident with the boundary of the collider or the cube's boundary, regardless of the visual interaction) is missing. Additionally, we utilized only one size of small collider. It would be valuable to explore whether there is a relationship between the size of the collider manipulated during the task and the observed perceptual after-effects. Future investigations are essential for improving our understanding of the interplay between visual and haptic characteristics during virtual object manipulation and its implications for human perception.

## Conclusion

The present study was conceptualized to measure possible after-effects in perception caused by incongruent visual or visual-haptic interactions with virtual objects. Specifically, the study examined how our perceptual system readapts itself following interactions with the environment under multisensory manipulations. We varied the size of a collider (the interactive space surrounding a virtual object) during a sensory-motor task and measured the after-effects of visuo-haptic incongruencies on size perception and the possible impairment in performance during the manipulation tasks. Findings revealed that visual incongruence during the interaction impaired performance in the manipulation task, regardless of the presence of haptic feedback. However, only in the group where small colliders were presented together with haptic feedback, the participants exhibited (as after effect) a shift in size perception, perceiving the cube as larger than their real size. The results were discussed in light of recent findings in visuo-haptic integration during multisensory information processing.

## Data Availability

Experimental data and R-code for reproducing the analysis are available in a OSF-repository: https://osf.io/qcmpr/.
